# The impact of visual complexity on perceived safety and comfort of the users: A study on urban streetscape of Sri Lanka

**DOI:** 10.1371/journal.pone.0272074

**Published:** 2022-08-09

**Authors:** L. W. G. Kawshalya, U. G. D. Weerasinghe, D. P. Chandrasekara

**Affiliations:** 1 Department of Architecture, Faculty of Architecture, University of Moratuwa, Moratuwa, Sri Lanka; 2 Faculty of Architecture, University of Moratuwa, Moratuwa, Sri Lanka; North Carolina State University, UNITED STATES

## Abstract

Increase in the variety of development in urban context has made it more complicated and complex for the users of public spaces. Absence of sufficient information to read the surrounding causes psychological anxiousness leading to perceived danger or discomfort for the urbanites. Consequently, perceived safety and comfort of the users is distinctively low in urban contexts, creating neglected and underused spaces. Complexity is one of the information processing variables as per Kaplan and Kaplan’s informational model which helps users to comprehend the surrounding environment. The streetscape plays a vital role in the daily movement patterns within the urban cities and is the transition boundary between the public and private realms. Visual complexity of these streets is a result of different configurations of elements within the urban areas. This research is conducted to ascertain the relationship between visual complexity levels of the streets with the perceived safety and comfort of the users. Shannon Diversity Index (SDI) and Fractal dimension analysis were conducted with 48 SVIs (Street View Images) selected within 1km radius of Colpetty junction, Colombo Sri Lanka covering all the possible compositions found within the context. The visual index data extraction had identified ten major components within the selected 48 SVIs. 78 subjective ranking responses for perceived safety, comfort (preference) and perceived complexity were collected from snowball sampling. Findings of the study revealed that perceived safety levels and preference scores for the SVIs are related to the Shannon Diversity Index calculation in an inverted ‘U’ shape where the highest and lowest SDI values are related with low preference scores and low safety levels. The SVIs with medium SDI values are perceived as the safest and most preferred by the users of urban streets of Colombo Sri Lanka. The SDI and fractal dimension values were significantly correlated with the perceived complexity scores of the users. The results of this study can be accommodated in the planning and designing of urban streetscapes of tropical climates for sustainable and friendly urban expansions.

## Introduction

With the developments in the urban contexts, most countries are facing different challenges in fulfilling the needs of their populations [1]. Along with the basic needs, the need for safety and comfortability in urban contexts has been emerging as a result of haphazard development with no consideration of the psychological needs of the users [2]. Perceived safety and comfort is a crucial aspect in the daily lives as it acts as a prerequisite for a quality lifestyle [3]. Scholars have proven that this fear of crime can be a result of a physical, economic, sociological, environmental or psychological factor [4]. This awkward and ominous feeling that crime or incivility yet to happen in the future might create no-go areas [5]. Emphasizing in this fact Blobaum and Hunecke [6] discusses Spielberger’s works where the perception of danger is considered as a cognitive appraisal of danger followed by an anxiety state reaction which will either be an avoidance behaviour, psychological defence or an application of a suitable coping mechanism. Thus, readdressing these public spaces as spaces which ensure the safety of the users will lead to successful urban management [7]. Unlike the relaxing spaces such as parks, squares and plazas, the time spent in the streets are basically utilized for movements, but the assurance of safety is important for the alleyways and specific spaces in the streets not to be underused or neglected [7, 8]. Streetscape is a requisite in the urban context as it promotes movement between the spaces and used by numerous users for the fulfilment of their daily needs [9]. The interactions with the elements and other people in surrounding are weak in the streets, still these quick interactions make a difference in effective utilizing of the space. The visual frameworks in urban streetscapes are complex due to the haphazard developments along the main facades. The correct interpretation of these complex visual arrays is indeed important in better designing with the assurance of perceived safety and comfort of the users [10]. The perception related studies have proven that the people perceive eighty percent of the information through visual perception [11, 12], while visual perception is equally important in perceived safety of the users [13–16]. The information obtained from the immediate environment was vital for the existence of the people in early ages of the evolution of human [17, 18]. An individual’s understanding of what is going around him will make him feel better since the individual can take necessary measures to secure if there is any possible threat in the surrounding. Alternatively an individual can perceive the surrounding freely and function well in that particular environment [18, 19]. Complexity is one of the information processing variables which is expressed as the diversity or the spatial combination of a space. Kaplan and Kaplan [18] has categorized complexity as an immediate information extracted through the surrounding for further exploration of the space. Complexity refers to the diversity, variety and the richness of the landscape with the combination of different patterns with the elements and features [20]. Kaplan [21] further elaborates that complexity directly corresponds to the number of things/ elements to be seen in a visual array and the level of information provided to the perceiver. Stamps [19] provides an explanation for ‘complexity’ after 30 years of Kaplan and Kaplans’ model as, “the number of elements of different varieties present in a scene providing information through their arrangements” [19]. With the idea that complexity is associated with levels of uncertainty, Berlyne [22] proposes that humans are happiest with medium levels of uncertainty in his ‘theory of aesthetics’. Ewing and Handy [23] discusses Amos Rapoport’s notion which elaborates that too little information can cause sensory distress while, too much information can cause sensory overload. Many scholars have concluded that the complexity of a streetscape is important for the users to find it appealing and user friendly [15, 24]. The live movements and activities of spaces also impacts on the perceptual aspect of the complexity in streetscapes [23, 25].

Many studies have focussed on the physical elements in the streetscape impacting directly on the visual complexity of the users. Ewing and Handy [23] further mentions Tony Neleseen’s assertation on the fact that if the same building design is repeated for more than three times in a building façade, then a boring and monotonous picture will be perceived. Thus, variety is always proposed for providing complexity. In terms of the building façade, texture, colour, and height can also impact on visual complexity [10]. Similarly, the shapes, articulation and decorations of buildings have also been studied for visual complexity [26, 27]. One major function of trees in urban context is to produce details which are no more there in the modern architecture [28]. The branching patterns of trees, the light penetration creating different aesthetics, the movements of the branches, the movements of the leaves play a special role in the process of visual complexity of the streets. The irregular shapes of the trees, the abstract formations of branches add complexity lacking in the urban context [28, 29]. In the assessment of visual complexity, the advertisements play a crucial role. A flawless signage can enhance the visual interest and make the street more welcoming and enhance the sense of place [30]. However these signage can be chaotic and cause problems for the pedestrian traffic [31]. The studies carried out separately for signages have proved that people prefer medium complexity in the visual qualities related to the advertisements [14]. The sky view of the streetscape is a major aspect in determining the complexity of the scenery. Although different moods of the sky (clear, cloudy, dark) will impact differently in the overall scenery, the sky view factor or the proportion of sky visible is an important aspect [32]. This is mainly considered in the perception studies as the proportion of sky view affected directly to the perceived openness of the streetscapes [33]. Apart from these static physical features, the human factor is also important in the addition of complexity to a scenery. It was also discovered that the most popular streets worldwide are the ones with frequent human interaction [29]. The perception related studies have concentrated more on the human factor as it provides a sense of belongingness, safety and comfortability for the users [34, 35].

The objective calculation of diversity is done in different methods including entropy calculations. From these, the most relevant and applicable objective measures to the urban sceneries and user experience perspective are Shannon Diversity Index (SDI), Simpson’s Diversity Index, Colour contrast and Fractal Dimension. Out of the listed, Shannon Diversity Index and Fractal Dimension have been used frequently in urban design related disciplines [15, 36, 37] and the same are being used as the objective measures in this study. Shannon Diversity Index, also known as Shannon Wiener Index and Shannon Entropy can be identified as a popular index to assess the diversity and variety of a physical context. This Index has been proposed initially by Claude Shannon in 1948 to quantify entropy or the surprisingness of a string of text [38, 39]. The base for this concept is, that higher the number of different letters the string makes, the more difficult it is to predict what is next. SDI quantifies uncertainty of the prediction of the identity/type of any random selection from the considered sample. Stamps [15] suggests the use of diversity indices as an image derived indicator for the assessment of complexity. With the use of Shannon Diversity Index (SDI), Stamps discovered a correlation between perceived diversity, pleasure and the SDI [15, 40]. The fractal dimension is used as the second objective measure of complexity in this study. The term ‘Fractal’ is derived from the Latin term ‘frangere’ and ‘fractus’ meaning fragmentes, meaning irregular and rough [41]. The term ‘fractal dimension was first used by Benoit Mandelbrot in his paper on ‘self-similarity’ where he assessed the fractional dimension as well [42]. Fractal dimension is a useful landscape metric in exploring irregularity and complexity of patterns in the landscapes [43]. This is frequently used in the landscape patch mosaic analysis carried out on the boundaries and the fragmentations [44]. This has been later extended to the urban form studies like land use analysis [36, 45]. Fractal analysis of urban form is carried out using three main methods, known as the perimeter-area related method, area-radius method and the box counting method [36, 46]. In the research related to the urban studies, box counting method is popular because of its simplicity and its effectiveness in terms of linearity, areas and volumes [37].

The primary aim of this study is to compare the levels of objective measures of complexity in the streetscapes of Sri Lanka with the perceived safety and comfort of the users; with the objectives; 1- Explore the complexity measures in the urban context related studies and perceptions of people, 2—Evaluate the complexity levels of the streetscape of Colombo, urban Sri Lanka (tropical context) using objective measures, and 3—Compare and evaluate the relationship between the explored complexity levels with the perceptual qualities assessed (complexity, safety, and comfort).

### Study area

**Fig 1:**
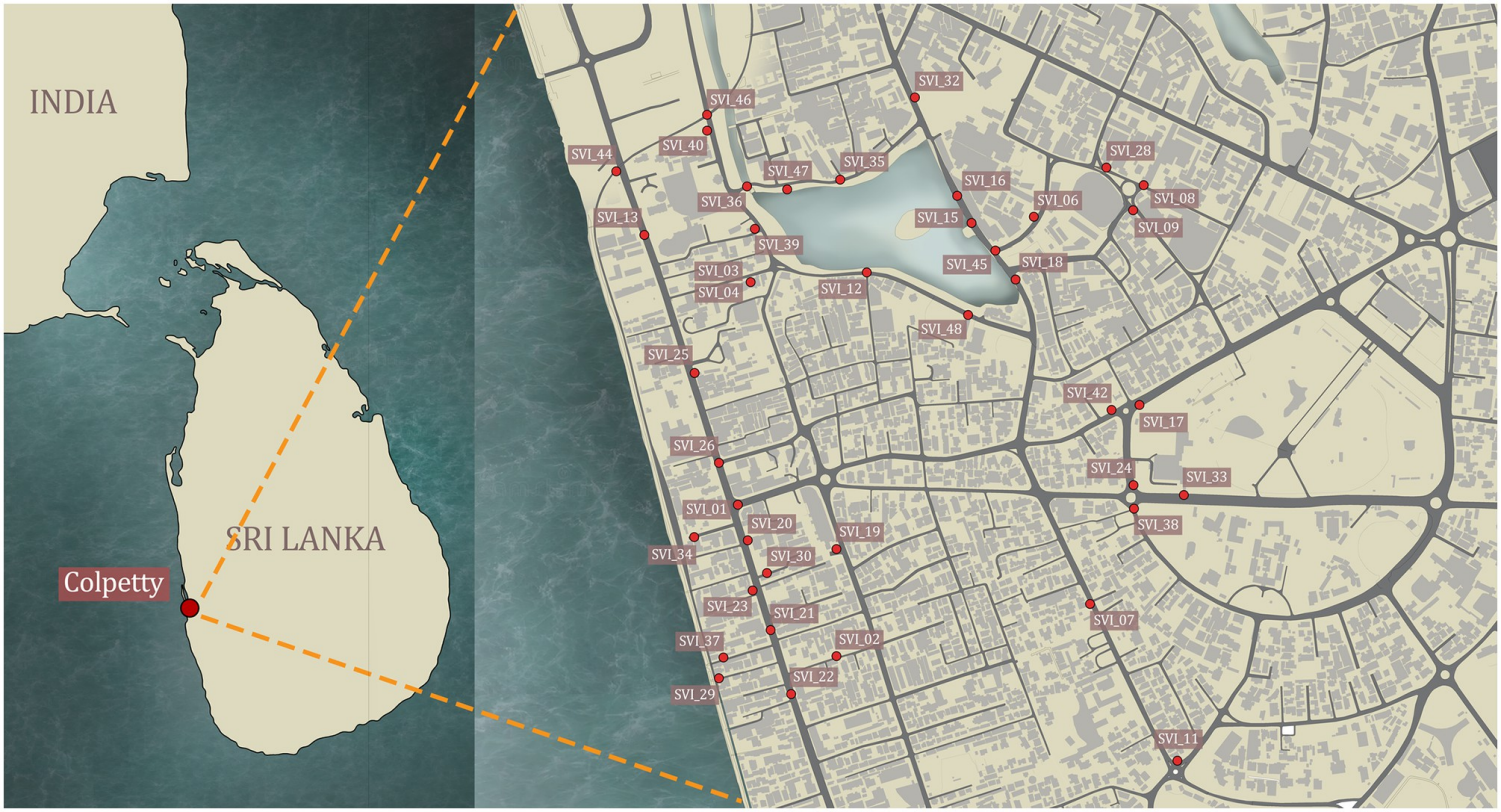
Case study locations of selected 48 SVIs—One kilometre radius from Colpetty Junction, Colombo Sri Lanka.

An area within one kilometre radius from Colpetty Junction, which is a major junction in the Central Business City of Colombo, Sri Lanka was selected as the case study area (Fig 1). Different streetscape configurations covering different identities across the urban context were chosen when selecting SVIs for the study. The selection process focussed more on the different arrangements on the street façade on either side of the road. The different levels of greenery, presence of water bodies, open spaces were considered when selecting the spaces. The selected area covers a part of the Viharamahadevi park, which is the oldest and the largest park within Colombo, a part of marine drive which opens to sea on one side and a part of Beira Lake which is a freshwater body within the boundary of Colombo. The selected area is situated in the commercial zone and most of the buildings are covered in cladding and the trees are limited in the road stretches. Enough green is present in the images selected near the Viharamahadevi park and Beira Lake. Most trees in images are drought tolerant evergreens which cope with the environmental conditions of Colombo. Advertisements displayed are mostly billboards where large sized ones are displayed at the high levels of buildings (rooftops) and medium sized ones adjacent to the roads in blank walls. Considering all these; a total of 48 SVIs (3508px × 1722px) were selected for the study with different configurations of the streetscape.

### Methodology

The Google Street View Images (SVIs) are used for analysis of the visual perspective of the users assessed through this study. Subjective perception has been examined frequently in research related to urban studies, where data collection in most cases have been carried out with person-to-person interactions [47]. But recently the use of SVIs in research has been more popular as it saves both time and resources [16, 32, 48–50]. Further, SVI assessments are always free from noise disturbance or any discomfort from temperature, humidity or wind, making it ideal for visual perception assessments [47]. It has been recommended to use SVIs for the studies on psychological state of the respondents and the characteristics of physical elements [51, 52]. The other studies which are concerned with feelings like safety have also incorporated SVIs for assessment [48, 53].

The street view photographs were directly taken from google street view which is the only source available in Sri Lanka. With the prevailing situation of COVID 19 in the country, the authors had to limit the SVI selection to the Google Street view imageries. The SVIs are composed of different street configurations (where both facades are buildings, trees in the median, roundabouts, facades with trees, facades open to sea) and different proportions of the components (buildings, vegetation, sky etc.). It should be noted that the author had no influence on the number of people or the number of vehicles in the SVI. All the SVIs were analysed in their original form as provided by the google street view. One main limitation in the selection process of the SVIs is that some spaces remained unclear with the interference of large vehicles in the proximity. So, the SVI selection was done removing these sceneries while maintaining the actual street configurations present in the area.

#### Objective measures of complexity

The horizonal perspective of the streetscapes provided by the SVIs make it more explicit to the users eye-level perception [48, 54, 55]. This study measures human perception through SVIs which are another form of human-centred streetscapes [56]. Strong statistical relationships between streetscape elements and different perception qualities have been identified in prior studies [23, 57, 58]. Many studies have used the view index of the elements for the assessment of complexity with the perspective images of the environment [33, 56, 59]. The view index was calculated with the pixel proportion of the selected feature to that of the total pixels of the SVI [59]. This was done through image segmentation which involves partitioning an image into multiple segments. The process of measuring the complexity of the SVIs are 1- selecting the SVIs with different configurations of streetscapes; 2- image segmentation with the identification of elements and components of the streetscape; 3- calculation of the visual indexes and; 4- calculating the SDI from the visual index data extracted (manual). The image segmentation of the SVIs were carried out with the use of AutoCAD software (developers: Autodesk, initial release– 1982). Ten separate segments were identified when partitioning the selected 48 SVIs, which are, building façade (BF), Vegetation (VEG), Sidewalk and related infrastructure (SW), Road (RD), Advertisements (ADV), Sky view (SV), Vehicles (VEH), Pedestrians (PED), Open spaces (OPN), and Water bodies (WAT).

The extracted visual indexes of the elements in streetscape are used for calculation of the Shannon Diversity Index, as follows; where M is the number of different Species and P*i* is the proportion of individuals belonging to the *i*^th^ species.


$$H=-\sum_{i=1}^{M}P_{i}\;\text{ln}\left(P_{i}\right)$$

In this case, ‘M’ is the number of different components/ elements of streetscape and the ‘*i’* is the visual index reading of the component/ element.

The fractal dimension of the SVIs were also assessed as a measure of complexity in this study. The fractal dimension assesses how detailed a fractal pattern of a scenery is. In most previous studies, fractal dimension was assessed with the patterns of dispersions of vegetation, waterbodies, and urban growth in the domain of planning and designing. This study accommodates the same and evaluates fractal dimensions of the selected SVIs considering each SVI as a complete urban patch. The box counting method is used for the extraction of Fractal dimension (D_b_). When an image of an urbanized patch is selected, first a single box (1×1) is taken so that it covers the entire area of the urban patch and the overall image of the city. The side length of the box r_1_, equals the side length of the basic grid, L. Then the number of boxes occupied by the urban patch N_1_ are counted. In this instance N_1_ = 1. The second step is to divide the grid by (2×2) and a total of 4 boxes cover the entire image. The side length of the box in the second instance is r_2_ = L/2. The number of boxes occupied by the urban patch are, N_2_. This process is repeated for *n* iterations where the number of boxes is recorded as N_n_. The box size r will be smaller with each step. Finally, the total area N_n_r_n_^2^ of the boxes occupies by the urban patch is calculated. The box counting fractal dimension is estimated as follows [37].


$$D_{b}=\underset{r_{n}\to \infty }{\text{lim}}\frac{log\;N_{n}}{log(\frac{1}{r_{n}})}$$

In practice log N_n_ and log (1/r_n_) is plotted in a scatter plot and the slope of the best fit curve is taken as the D_b_ for the considered image. It is estimated as follows [37].


$$\text{ln}\;N\left(r\right)+D_{b}\;\text{ln}\left(\frac{1}{r}\right)+b_{1}=0$$

**Fig 2:**
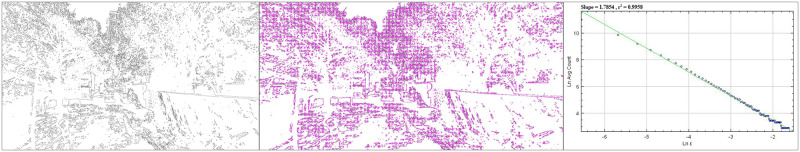
(1) Fractal dimension calculation steps in ImageJ Fractlac. (2) box counting image presentation (3) best fit line with the scatterplot.

The study accommodated the ImageJ software (initially developed by Wayne Rasband (1997), United States) for the analysis of the SVIs. ImageJ is a free software which calculates a variety of statics related to the pixels of imagery [60]. The Fractlac plugin (Author A Karperien 2007), Australia which is specially developed for the analysis of the fractal dimension D_b_ is used for the analysis. Fig 2 represents the outputs from the software.

#### Subjective measures of complexity

Apart from the objective measures of Complexity, subjective components were also assessed within the three perceptual qualities of perceived safety, perceived comfort, and perceived complexity. The selected 48 SVIs were assessed, being ranked based on the three perceptual qualities by 78 urban users. The users were selected through snowball sampling method, where individuals involved in design related disciplines like architecture, landscape architecture, urban planning, urban design etc. were selected. This user group was identified as ideal for this study, with their knowledge to identify and distinguish between the visual qualities of urban sceneries. The distributed questionnaire (online–maximum interaction the authors could afford at the time of COVID 19) requested the users to provide their ranking for each SVI in a 1–7 rating scale. This rating scale was used as all the respondents were knowledgeable about the visual aspects of urban sceneries and were capable of judging each SVI from a considerable range of choices. The users/ respondents were asked to imagine that; they are at the location (in a common perspective–not specifically as a pedestrian or a driver) and looking at the perspective view of the streetscapes. They were asked to rank their level of safety, preference, and the level of complexity. The preferences were assessed with the intention of analysing the sense of comfort of respondents with the scenery. As far as psychological aspects are concerned, application of SVIs was considered best as the users will not be affected by microclimate and other environmental conditions of the physical setting.

Statistical analysis of the collected data was carried out using the SPSS software (Statistical Package for Social Sciences–developed by Norman H. Nie, Dale H. Bent, C. Hadlai Hull, initial release:1968, Country: USA). Non-parametric tests were carried out for the analysis of the relationship between the components of streetscape and the explored perceptual qualities of Perceived safety, preference, and perceived complexity. The view indexes of the 10 components (BF, VEG, SW, RD, ADV, SV, VEH, PED, OPN and WAT) of the streetscape were divided into several groups to assess responses to the perceptual qualities. After examining all 48 SVIs, the most frequently occurring features were categorized into three levels as low, medium, and high according to the visual indexes calculated. The building facades (BF), vegetation (VEG), sidewalks and other infrastructures (SW), road (RD), sky view (SV) and vehicles (VEH) were categorized into three different levels as low, medium, and high. These components were categorized as the variation of these components in the SVIs is relatively high compared with the other components. The other special features like advertisements (ADV), pedestrians (PED), open spaces (OPN) and waterbodies/ water features (WAT) were categorized as present and absent accordingly. Then these were analysed with the non-parametric tests, Kruskal-Walli’s H (three independent categories) test and Mann-Whitney U test (two independent categories) using the SPSS software.

## Results and discussion

The Mann-Whiteny U test results for the gender analysis with the perceptual qualities (safety, preference, and complexity) revealed that there is a significant difference in the responses between the male and female with perceived safety while perceived complexity and preference showed no difference across the categories. The mean ranks between the categories represents that the safety scores of the males are higher than that of the females [61]. The overall analysis of the SVIs represented view indexes of different elements of the streetscape as; 25.71% of building facades (BF), 16.76% of vegetation (VEG), 8.28% of sidewalks and other related infrastructure like utility lines, utility boxes etc (SW), 23.72% of road (RD), 1.74% of advertisements (ADV), 17.74% of sky view (SV), 3.26% of vehicles (VEH), 0.20% of pedestrians (PED), 1.66% of open spaces (OPN), and 0.93% of waterbodies (WAT).

The separate visual indexes of the components in the 48 SVIs are reported in Table 1.

**Table 1:** View indices of the street elements for the selected 48 SVIs. (Values are presented as percentages).

ID	BF	VEG	SW	RD	ADV	SV	VEH	PED	OPN	WAT
**SVI _01**	34.30	0.36	3.49	25.86	8.61	19.80	7.38	0.19	0.00	0.00
**SVI _02**	74.98	3.30	0.14	7.95	0.00	8.58	5.03	0.02	0.00	0.00
**SVI _03**	40.48	29.48	6.99	12.48	0.00	6.43	4.14	0.00	0.00	0.00
**SVI _04**	23.31	54.56	4.18	14.89	0.00	2.24	0.45	0.37	0.00	0.00
**SVI _05**	32.90	18.13	6.32	23.49	0.00	19.03	0.13	0.00	0.00	0.00
**SVI _06**	6.06	26.56	11.24	33.42	7.05	11.65	3.86	0.16	0.00	0.00
**SVI _07**	11.34	34.62	7.06	21.12	0.00	14.32	0.07	0.00	11.47	0.00
**SVI _08**	39.06	30.67	3.80	19.36	1.65	4.56	0.90	0.00	0.00	0.00
**SVI _09**	41.61	23.12	4.91	27.47	0.00	2.90	0.00	0.00	0.00	0.00
**SVI _10**	33.70	3.33	6.74	20.27	8.09	20.82	6.95	0.09	0.00	0.00
**SVI _11**	9.17	36.98	7.07	33.16	1.28	10.03	2.30	0.00	0.00	0.00
**SVI _12**	19.92	11.48	18.76	22.48	0.00	19.33	1.89	0.00	0.00	6.14
**SVI _13**	32.69	12.56	16.79	17.88	0.00	19.67	0.21	0.01	0.00	0.19
**SVI _14**	40.58	0.17	3.89	29.85	0.00	21.66	3.48	0.37	0.00	0.00
**SVI _15**	8.81	26.28	14.08	27.88	0.11	18.90	2.43	0.11	0.00	1.41
**SVI _16**	3.76	13.25	15.59	19.19	0.33	41.29	1.54	0.11	0.00	4.94
**SVI _17**	9.69	29.38	9.28	35.93	1.37	12.28	0.66	0.06	1.36	0.00
**SVI _18**	2.80	34.58	13.38	16.93	0.51	18.12	9.77	0.00	1.26	2.65
**SVI _19**	28.98	3.16	6.39	19.36	9.99	16.90	11.48	1.51	2.23	0.00
**SVI _20**	29.46	6.18	5.21	34.32	2.15	15.84	6.44	0.40	0.00	0.00
**SVI _21**	37.23	4.39	4.24	26.30	5.51	12.76	9.25	0.33	0.00	0.00
**SVI _22**	40.95	0.38	4.95	12.35	1.57	24.63	13.95	0.00	1.22	0.00
**SVI _23**	31.51	12.90	9.13	30.17	2.91	12.04	0.85	0.50	0.00	0.00
**SVI _24**	4.38	35.48	3.26	29.25	10.76	11.25	0.34	0.37	4.91	0.00
**SVI _25**	26.73	7.73	11.88	22.58	0.00	30.99	0.10	0.00	0.00	0.00
**SVI _26**	35.17	21.48	13.14	15.48	0.16	14.07	0.46	0.06	0.00	0.00
**SVI _27**	31.81	6.53	5.90	32.26	3.91	16.94	1.77	0.11	0.00	0.76
**SVI _28**	24.39	24.11	4.55	16.92	1.47	11.74	0.20	0.05	16.57	0.00
**SVI _29**	6.15	0.36	6.61	29.82	2.50	47.17	3.00	0.00	0.00	4.39
**SVI _30**	59.95	1.40	1.31	12.03	0.00	4.32	20.41	0.57	0.00	0.00
**SVI _31**	15.92	6.04	5.40	42.38	2.90	22.70	3.96	0.00	0.70	0.00
**SVI _32**	32.58	10.58	6.84	20.97	0.53	21.23	3.05	1.16	3.05	0.00
**SVI _33**	11.61	40.60	1.97	40.24	0.00	5.25	0.33	0.00	0.00	0.00
**SVI _34**	16.66	3.71	8.32	14.45	0.27	47.12	9.10	0.11	0.00	0.26
**SVI _35**	29.30	13.52	11.19	14.34	0.00	28.06	0.00	0.01	0.00	3.58
**SVI _36**	6.53	19.04	17.31	22.20	0.00	25.95	0.00	0.00	0.00	8.98
**SVI _37**	66.38	0.00	10.73	0.05	0.00	10.29	6.99	0.00	5.35	0.21
**SVI _38**	5.49	19.43	3.50	38.66	5.83	15.92	0.60	0.00	10.57	0.00
**SVI _39**	14.68	13.68	10.38	28.82	0.00	31.35	0.14	0.00	0.00	0.94
**SVI _40**	18.30	28.81	9.52	21.41	0.00	19.80	0.04	0.00	0.00	2.12
**SVI _41**	24.31	8.43	10.50	20.47	2.42	28.45	3.92	1.24	0.26	0.00
**SVI _42**	24.14	12.81	7.55	28.89	0.54	20.66	5.19	0.22	0.00	0.00
**SVI _43**	48.95	10.09	5.45	25.12	0.00	8.58	1.12	0.32	0.38	0.00
**SVI _44**	35.21	17.79	18.08	22.67	0.26	5.74	0.24	0.00	0.00	0.00
**SVI _45**	13.61	18.20	11.72	32.61	1.06	19.17	1.85	1.19	0.00	0.61
**SVI _46**	19.00	16.10	9.98	28.40	0.00	20.58	0.06	0.00	4.72	1.15
**SVI _47**	27.45	22.87	8.91	18.78	0.00	12.82	0.00	0.00	4.86	4.30
**SVI _48**	1.89	30.10	9.71	27.61	0.00	17.74	0.26	0.01	10.69	2.00

**Fig 3:**
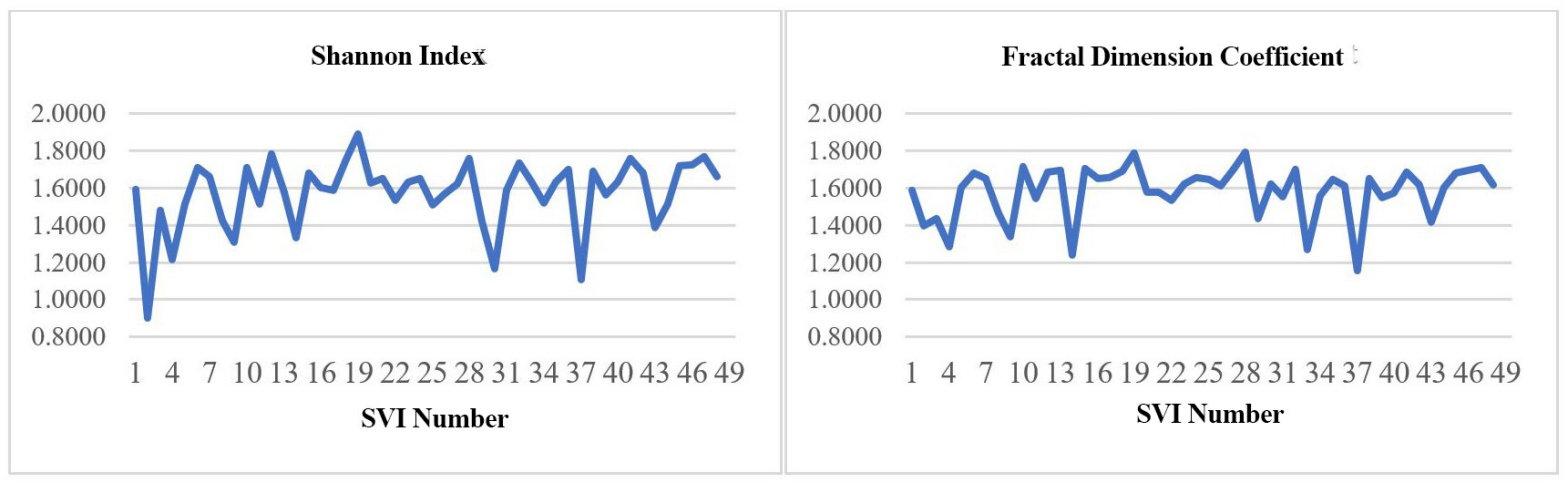
Shannon Diversity Index (SDI) and fractal dimension coefficient variation across the 48 SVIs.

With the above view indexes, the SDI and the fractal dimension coefficients were calculated for the 48 SVIs. The SDI values for the 48 SVIs ranged from 0.9012 to 1.8888 and the fractal dimension coefficients varied from 1.1548 to 1.7891 as shown in the Fig 3.

The subjective ranking scores were collected for all the 48 SVIs from the respondents. 55% of the respondents were male while 45% of the respondents were female. Majority of the respondents (73%) were between 19–28 years. 46% are residing in the urban areas while 42% are residing in the suburbs while remaining 12% are from rural areas. 79% of the users frequently use the urban streets in their day-to-day movements within the city.

The view index values calculated for the building facades (BF) of SVIs were categorized into three levels as low, medium, and high. The Kruskal Walli’s H test result provided strong evidence of a significant difference between the perceived safety levels for the three groups (p<0.05). Then the post hoc test was carried out to explore pairwise comparison of the three groups. This comparison was done through an adjusted significance value by the Bonferroni correction for multiple tests. The results showed that the perceived safety between medium–high levels of building facades are similar (p>0.05) and other pairwise comparisons (high–low and medium–low) are significantly different (p<0.05). According to the average mean ranks, highest perceived safety is seen with low building façade levels (2210.61) when compared with medium (1662.83) and high levels of building facades (1744.06) in the SVIs. Similar results were shown for the preference as well, where users have preferred low levels of the buildings in their vicinity. When considering visual complexity, high mean ranks were seen in the medium levels of the building facades. According to Ewing & Handy [23], increase in the number of the buildings have contributed towards increasing perceived complexity of the users.

Similarly, Kruskal Walli’s H test was carried out for the permeability levels of the building facades. Here the author categorized the 48 SVIs as low, medium, and high permeability. Low permeability was assigned to the blind walls and adjacent facades where no activity is visible. Medium level permeability was assigned to the facades where there are certain activities or low-level walls to the private properties allowing a medium level visual access through the façade. High permeability was assigned to the facades with shop fronts, balconies or similar elements which promote active participation of the users creating dynamic facades. The results depicted that there is a significant difference in the scores for safety, preference, and complexity across the levels of permeability of the street facades. Higher mean ranks for all three perceptual qualities were associated with high and medium levels of permeability (adj. P>0.05 for the high and medium for all 3 perceptual qualities) thus demonstrating that active facades are safer and preferred by the users. This is basically a result of the concept of copresence or the eyes on the street which is a frequently discussed in the domain of Environmental Psychology [7, 62–65]. It was revealed in a prior study. that blind walls alongside of the urban streets are negatively related with the sense of safety of the users. Thus, active facades in the streets are an assurance of possible help in case of a probable threat/ crime [66, 67].

In the domain of Environmental Psychology, Sociology, Architecture, Landscape Architecture and Urban Planning, the relationship between the vegetation and the perceptual qualities of the users are explored. These studies inquire the basic properties associated with trees and the spatial qualities associated with vegetation. Here the results from non-parametric Kruskal Walli’s H test suggests the medium level of vegetation is more biased towards high perceived safety levels. The SVIs where medium level vegetation is present has been assessed by the authors and the most common feature seen in all the SVIs were that none of these vegetations (medium level) had obstructed the views. These were located either in the background or midground of the SVIs. There was no visual obstruction with these medium level vegetation unlike the high levels of vegetation. Similarly, Jorgensen et al [68] has found that dense vegetation is perceived unsafe because of obstructed visual accessibility. The SVIs categorized under the low levels of the vegetation includes the well-maintained vegetation found in the junctions or planted shrubs in the median. It has been proven that the visual access and penetration is important for the preference in forest settings [69]. Unlike a park or forest, a streetscape should facilitate visual penetration as it is highly important for the safety of both motorists and the pedestrians, and it is adequately represented by the results. When considering the Perceived complexity related with vegetation, the complexity increases with the vegetation as a result of the increasing ‘shape defining points’ which is visually associated with irregular dispersion of the tree canopy and the branching patterns of trees [40]. The low levels of vegetation do not influence these shape defining points as it is mostly the shrubs and low-level plants in the visual frame. But medium to high level vegetation can make the most significant impact on perceived complexity with the shape defining points of its irregular growth. The results represent that the users perceive medium to high vegetation levels as more complex than low vegetation levels. Similar to the findings by many other scholars, the result of this study proves that high visibility of green is more preferred by the users specially in urban areas [48, 56, 70–72].

Kruskal Wall’s H test was repeated for the sidewalk (SW), road (RD), Skyview (SV), and Vehicles (VEH). High mean ranks for perceived safety and preference were recorded in the medium levels of sidewalk infrastructure (SW) and perceived complexity was interconnected with high levels of infrastructure in the sidewalk. For the road (RD) covering the SVIs, high mean ranks for the perceived safety and preference were associated with high coverage of the road areas in the SVIs. These roads cover a large portion of the SVIs creating a prospect in the view of the carriageway which is directly associated with the sense of safety [33]. These road views are mostly empty with low number of vehicles creating it an open space in the middle of the SVIs. The perceived complexity was associated with medium levels of the view of the road, where the view is obstructed with other elements in the vicinity. There was no significant difference across the three different levels (p>0.05) for the sky view (SV) where the sky view area did not affect the perceived safety of the users. But medium level of sky view was chosen as the best level by the users for perceived complexity. Zeng et al. [32] demonstrates that sky view index is associated with the perception of openness of a space. A study carried out by Qiu et al. [56] claims that sky view index is associated with high levels of safety and higher levels of feeling of enclosure. But the current study analysis does not have enough data to prove or disprove this claim. When considering the vehicles in the SVIs, medium to low levels of vehicles were more favoured by the users for perceived safety and preference. Having vehicles at the road implies, copresence in the surrounding. Too many vehicles in the adjacent lanes provides discomfort visually as well as physically. Sri Lanka being a tropical country, these impacts increase with the nature of urban spaces and harsh environmental conditions.

The street elements as advertisements (ADV), open spaces (OPN), water bodies (WAT) and presence of pedestrians (PED) in the SVIs were categorized into two levels as present or absent. Mann Whitney U test was used to analyse these elements. According to the results, presence of advertisements showed a significant difference (p<0.05) between all three perceptual qualities where high perceived safety levels and perceived complexity levels. The absence of advertisements was preferred by the users as these adverts impact negatively and enhance the chaotic nature of the urban streets. According to findings of numerous research, the presence of advertisements results in decrease of the sense of safety of the users [7, 73, 74]. As the current study contradicts this finding, a close analysis was conducted for each SVI containing the advertisements. Close analysis on the presence of advertisements or billboards on the street exposed that all these advertisements had no impact on the visual arrays. Most of these were displayed against the blind walls along the street facades. But this presence has resulted in increasing perceived complexity of the streetscape. The outcome confirms with findings of previous research according to which, the presence of billboards, advertisements and graffiti in urban context increases the disorder of a scenery making it more complex to the eye [7, 31, 56].

The open spaces (OPN) considered in the study are basically the parks adjacent to the street (Viharamahadevi Park, Colombo), water bodies and open parking spaces along the street. According to Mann Whitney U test results, the open spaces have had a significant impact on the three perceptual qualities (p<0.05). With close examination on each open area in the selected SVIs, it can be concluded that people feel anxious with the open areas adjacent to the sidewalk with no coverage. People feel safer and comfortable when there is an adjacent building façade present with the sidewalk. A study done by Anderson and Stokes [75] on perceived safety and attractiveness of parking lots has discovered that proximity to buildings is positive related to security of the users. When considering perceived complexity, the presence of open spaces has caused an increase in the complexity. The presence of open spaces has led to better views of the trees and other elements increasing the visibility of the shape defining points where this is directly impacting on the perceived complexity. The SVIs were then categorized with the visibility of the water bodies in the view and there was no significant difference for perceived safety and complexity (p>0.05). But the preference of the users had recorded high values for the SVIs with view of water bodies. It strengthens the argument forwarded by many scholars that urban users enjoy sights of blue in the vicinity as it provides the feeling of relaxation in the midst of chaotic urban settings [64, 73, 74, 76]. The presence of pedestrians (PED) in the SVIs showed no significant difference (p>0.05) for all three perceptual qualities. The presence of pedestrians in the SVIs were not very noticeable and this can be the reason for absence of any difference across the two levels.

Spearman’s Rank correlation analysis was carried out with the perceptual quality scores across the 48 SVIs and the calculated measures of complexity. The results are represented in the Table 2.

**Table 2:** Spearman’s rho values for the measures of complexity and perceptual qualities.

		Shannon Index	Fractal Dimension
**Spearman’s rho**	Perceived Safety	-.175	-.021
	Preference	-.193	-.207
	Perceived complexity	**.841** **	**.661** **

**. Correlation is significant at the 0.01 level (2-tailed).

*. Correlation is significant at the 0.05 level (2-tailed).

**Fig 4:**
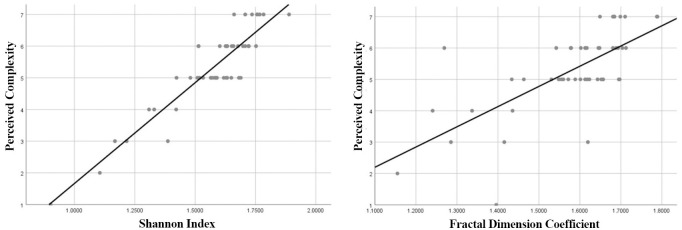
Linear relationship between the perceived complexity with Shannon Diversity Index (SDI) and fractal dimension coefficient.

The results show a highly significant values for perceived complexity with objective measures of complexity with a correlation coefficient of r_s_ = .841, p<.000 for the Shannon Index and a correlation coefficient of r_s_ = .661, p<.000 for the Fractal Dimension. This simply proves that there is a significantly strong relationship between what is perceived by the users and the objective measures of the complexity. When the Shannon Diversity Index (SDI) value increases for a given imagery, the users will experience a feeling of high visual complexity and vice versa (Fig 4).

**Fig 5:**
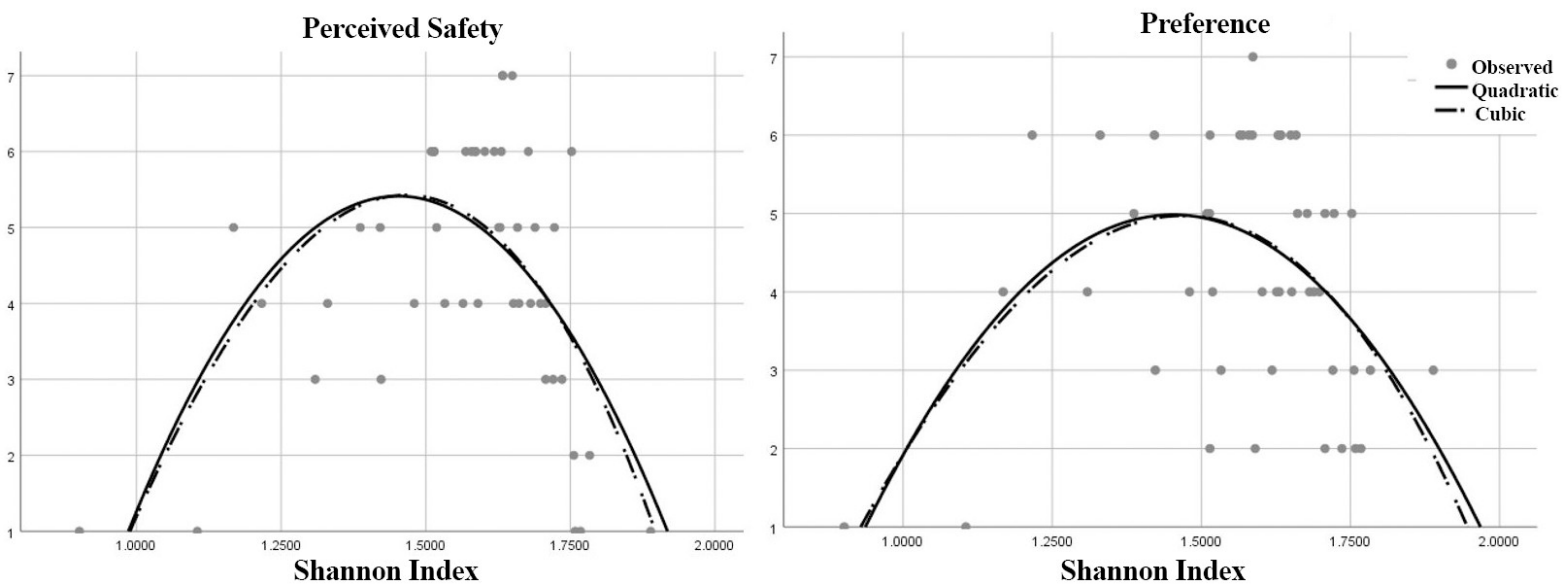
Non-linear relationship between the perceived safety, preference with Shannon Diversity Index (SDI).

Fig 5 shows analysis of perceptual qualities with the aid of Shannon Diversity Index (SDI). The result shows a non-linear relationship between the variables. The curve estimation line through the data set depicts a significant bell-shaped relationship (cubic equation (R^2^ = 0.439, p<.000)) with the two variables. A similar relationship was seen between SDI and preference with a possible cubic equation (R^2^ = 0.271, p<.001). It illustrates that, higher levels and lower levels of complexity are associated with low preference and low safety perception. Medium levels of complexity is highly preferred by the users and they feel more safe and comfortable in medium visual complexities [40, 77]. Similar behaviour is seen between the perceived complexity levels and the perceived safety levels with a possible quadratic equation with (R^2^ = 0.323, p<0.001) and between the perceived complexity levels and preference with a possible cubic equation of (R^2^ = 0.516, p<0.000).

The measure of fractal dimension does not show any significant relationship with the perceived safety scores or with the preference score. The Spearman’s rho (Coefficient of correlation) (r_s_) of the perceptual qualities shows a statistically significant and a strong positive relationship of r_s_ = .651, p<.000 with preference. The increase in safety to a particular view will increase the preference of the users.

## Conclusion

The information model of Kaplan and Kaplan (1989) asserts that information extraction from the immediate surrounding directly relates to the feeling of safety of an individual. The complexity, diversity and spatial configuration helps in extracting the immediate information from an environment. Research explorations of the contribution of visual aspects for the information perception of an environment is frequently seen in Environmental Psychology and other related domains. This research is an attempt at assessing visual complexity of the streetscape and has endeavoured to explore the quantitative measures of complexity with the perception of users. The location of study, Colombo, in tropical Sri Lanka unveils different dimensions of complexity with the perceptual qualities assessed. The selected 48 SVIs have recorded significant complexity with two objective measures of visual complexity: Shannon Diversity Index (SDI) and the Fractal Dimension analysis.

The results of non-parametric tests depict that, high levels of safety and preference were associated with the low density of building facades (BF) with high to low permeability levels. Furthermore, high to medium levels of visual permeability of the building facades (facades with shop fronts, balconies or other amenities which promotes activities) has accounted for the increase in safety, preference, and complexity of the users. Medium levels of vegetation (VEG) were selected as the best scenario for the perceived safety which allows visual penetration to the adjacent spaces. The results further proves that high levels of greenery and presence of water was preferred which counts for the visual and physiological comfort of the users. Medium to low levels of vehicles were preferred to be safer with ‘eyes on street’ or the copresence of other users in case of a possible threat. Too many vehicles have accounted for the visual and physical discomfort of the users. Moreover, both the measures of objective complexity (SDI and Fractal Dimension) showed very significant association with the perceived complexity of the users. Further analysis on the connection between SDI and perceived safety and preference revealed that there is non-linear relationship between them. The curve estimation analysis showed a clear result which illustrates low levels of perceived safety and preference is associated with both low and high levels of SDI. The medium levels of SDI have been perceived as most safe and most preferred by the users of urban streets of Colombo Sri Lanka, illustrating an inverted U shape relationship. The perception of the users is often overlooked during the design and implementation stages of most of urban development projects. But inputs from the prospective users will enable urban designers determine the most liveable spaces which are preferred by the urbanites. The perceived safety and comfort are a vital component to be fulfilled in urban spaces as the assurance of this will determine whether spaces will be used actively or not. The exploration of the relationship between the measures of complexity with perceived safety and comfort provide insights into user perception of the streetscape. This study further proves that the objective measure of complexity reflects the perceived (subjective) complexity levels of the users. Through this research, it has been established that urban street configurations which is visually perceived as medium levels of complexity will be ideal for assuring the perceived safety and comfort of the users. The correct utilization of these results will lead to a sustainable urban space designing. The expansion of the research in other cities can provide more insights to the results and a valid comprehensive model can be developed. Only a limited amount of similar research has been carried out in the tropics and this study can be an initiation for similar research which can further expand with the consideration of other unique factors in the tropics.

## Supporting information

S1 Data (XLSX)
